# Health related quality of life in multimorbidity: a primary-care based study from Odisha, India

**DOI:** 10.1186/s12955-019-1180-3

**Published:** 2019-07-05

**Authors:** Sanghamitra Pati, Subhashisa Swain, J. André Knottnerus, Job F. M. Metsemakers, Marjan van den Akker

**Affiliations:** 1 ICMR Regional Medical Research Centre, Bhubaneswar, Department of Health Research, Chandrasekharpur, Indian Council of Medical Research, Bhubaneswar, Odisha 751023 India; 2 Indian Institute of Public Health, Bhubaneswar, Public Health Foundation of India, And School of Medicine, University of Nottingham, Clinical Sciences Building, City Hospital, Nottingham, UK; 3 Dept. Family Medicine, School Caphri, Maastricht University, Maastricht, The Netherlands; 4 Academic Centre of General Practice/Department of Public Health and Primary Care, KU Leuven, Leuven, Belgium; 5 Institute of General Practice, Johann Wolfgang Goethe University, Frankfurt am Main, Germany

**Keywords:** HRQoL, Quality of life, Multimorbidity, Multiple chronic conditions, Primary care, India

## Abstract

**Background:**

Multimorbidity, the coexistence of two or more chronic conditions is increasingly prevalent in primary care populations. Despite reports on its adverse impact on health outcomes, functioning and well-being, it’s association with quality of life is not well known in low and middle income countries. We assessed the health-related quality of life (HRQoL) of primary care patients with multimorbidity and identified the influencing factors.

**Methods:**

This cross-sectional study was done across 20 public and 20 private primary care facilities in Odisha, India. Data were collected from 1649 adult out-patients using a structured multimorbidity assessment questionnaire for primary care (MAQ-PC). HRQoL was assessed by the 12-item short-form health survey (SF-12). Both physical (PCS) and mental components scores (MCS) were calculated. Multiple regression analysis was performed to determine the association of HRQoL with socio-demographics, number, severity and typology of chronic conditions.

**Results:**

Around 28.3% [95% CI: 25.9–30.7] of patients had multimorbidity. Mean physical component scope (PCS) and mental component score (MCS) of QoL in the study population was 43.56 [95% CI: 43.26–43.86] and 43.69 [95% CI: 43.22–44.16], respectively. Patients with multimorbidity reported poorer mean PCS [43.23, 95% CI: 42.62–43.84] and MCS [41.58, 95% CI: 40.74–42.43] compared to those without. After adjusting for other variables, morbidity severity burden score was found to be negatively associated with MCS [adjusted coefficient: -0.24, 95% CI − 0.41 to − 0.08], whereas no significant association was seen with PCS. Hypertension and diabetes with arthritis and acid peptic diseases were found to be negatively related with MCS. Within multimorbidity, lower education was inversely associated with mental QoL and positively associated with physical QoL score after adjusting for other variables.

**Conclusion:**

Our findings demonstrate the diverse negative effects of multimorbidity on HRQoL and reveal that apart from count of chronic conditions, severity and pattern also influence HRQoL negatively. Health care providers should consider severity as an outcome measure to improve QoL especially in individuals with physical multimorbidity. Given the differences observed between age groups, it is important to identify specific care needs for each group. Musculoskeletal clusters need prioritised attention while designing clinical guidelines for multimorbidity.

**Electronic supplementary material:**

The online version of this article (10.1186/s12955-019-1180-3) contains supplementary material, which is available to authorized users.

## Introduction

Over the past decades, the combination of improved living conditions, better prevention and management of infectious diseases, continuous medical developments, along with the overall aging of the population, have substantially increased the prevalence of chronic non-communicable diseases (NCD) globally [1, 2]. Consequentially, multimorbidity, the co-occurrence of two or more chronic diseases, is being frequently observed in individuals and can be regarded as an “emergent” health problem [3]. A study conducted by Barnett has reported the prevalence of multimorbidity to be around one third [4]. Studies based on secondary data and surveys have also reported multimorbidity to be a prevalent phenomenon in low and middle income countries (LMIC) given their rapid demographic transition [5, 6]. However, until now, the corpus of research output on multimorbidity is disproportionately deficient in LMIC with scarce evidence from primary care populations reflecting the existence of the ‘inverse care law’ or the availability of appropriate health care varying inversely with the need for it in the population served, thus perpetuating health inequity [7].

Chronic diseases per se persist for an extended period and affect a person’s ability to function normally. Thus, multimorbidity has the potential to induce profound negative effects on a person’s quality of life and wellbeing [8–10]. Patients with multimorbidity frequently present with lower functional capacity, higher health care utilisation, increased levels of polypharmacy, and mortality rates in addition to the risk attributable to individual disease [11, 12].

The health-related quality of life (HRQoL), a composite index of subjective wellbeing, is currently being widely used as a key health outcome measure [13]. HRQoL is a patient-centred outcome that assesses the impact of health conditions on daily living, based on the self-perception of the individuals, and considers their social and cultural context. Available evidence demonstrates that HRQoL is negatively associated with multimorbidity in hospital, outpatient clinic and community settings [14, 15]. While several studies have described the impact of multimorbidity on HRQoL, using the simple count of chronic conditions in patients as a measure, the effect of severity and combinations of different chronic conditions on HRQoL level is not fully known [14, 15]. Further, compared to the western countries, there is limited understanding regarding the level, and contributing factors of HRQoL in multimorbidity in LMIC. Our systematic review has revealed the conspicuous absence of any study from South Asia describing the effect of multimorbidity on HRQoL among primary care patients [16].

With its relatively recent entry, multimorbidity still is an under-studied entity in India, constituting a critical knowledge chasm for a country witnessing an unprecedented high rise of NCDs [17, 18]. Few studies based on health surveys have reported the prevalence of multimorbidity to be ranging from 8.9 to 57% and suggested that elderly people were more likely to have inferior HRQoL [5, 19, 20]. However, these findings have limited generalisability being based on secondary data and a population with a small number of chronic morbidities included. In India, since the majority of multimorbidity patients are seen in primary care routinely [21], it is necessary to have information on the status of HRQoL in these patients, contributing factors and the extent to which HRQoL is affected when the number, pattern and severity of conditions change [20].

In view of the above, we undertook a study to assess the health-related quality of life of a primary care patient population and its sociodemographic correlates. We further determined the effect of count, type, severity and combinations of chronic conditions on HRQoL within multimorbidity. We expect that our findings would contribute towards identifying subgroups having differentially impaired quality of life within multimorbidity and to designing specific care plans.

## Methods

### Study sample

We carried out a cross-sectional study in 20 public and 20 private primary care facilities in 10 districts of Odisha state, India. The facilities were selected using a multistage sampling process. We included private clinics along with public primary care as considerable proportion of population in India consult private practitioners for health care [21]. Details of the sampling methodology have been published elsewhere [6].

We did not have any studies on prevalence of multimorbidity from India, the required sample size was calculated based on our pilot study while validating the multimorbidity assessment tool. Considering that 23% of patients attending primary health care settings have multimorbidity [22], a sample of at least 1456 was needed to estimate this level of prevalence within relative precision error of 12.5% and design effect of 1.7 for multilevel cluster design. After accounting for 13% non- response based on our pilot experience [22], the final required sample size was 1670. It was decided to divide this number equally between private and public primary care facilities.

### Data collection

All adult patients (aged 18 years and above) attending the sampled facilities who met the predefined inclusion and exclusion criteria were approached through systematic sampling method. Comatose patients were excluded. Selected patients were interviewed using a structured multimorbidity assessment questionnaire for primary care (MAQ-PC) in local language, by four trained nurses. Given the absence of a standardised uniform tool to assess multimorbidity in primary care, we had previously developed MAQ PC through an iterative process and evaluated its reliability and internal consistency. The questionnaire entails assessment of self-reported multimorbidity in which respondents reacted to a list of common chronic diseases. The development and validation of the tool and its components have been detailed in a previously published article [22]. The socio-demographic section of the questionnaire elicited information on age, gender, place of birth, residence, ethnicity, religion, educational level, marital status, poverty status as per state guideline, current housing and household composition.

The multimorbidity assessment section of the questionnaire elicited the presence of any of the 18 listed self-reported chronic diseases. The list of chronic conditions was prepared based upon the findings from our pilot study, extensive literature search, chart review and expert consultations [22, 23]. There were open options for additional conditions if any to be added by the patient. To evaluate depression, along with physician-diagnosed self-report, we included the Patient Health Questionnaire (PHQ-9) validated for the Indian population [24]. The prevalence of diagnosed depression in primary care has been reported to be extremely low in India; moreover, depression has fluctuating symptoms and is amenable to long-term treatment; therefore, we assessed current depressive symptoms with a short and already validated screening test rather than relying only on a diagnosis of depression [25, 26].

We additionally assessed severity of each of the reported chronic illnesses. Respondents rated each condition on a 5-point scale from 1 (interferes with daily activities “not at all”) to 5 (interferes with daily activities “a lot”). The total score representing level of morbidity (which we also refer to as severity burden score) was the sum of the weights of the diseases by the level of interference for each condition [27].

The SF-12 questionnaire was used to measure participants’ functional status and health-related quality of life. The SF-12 generates eight subscales which measures different dimension of health [28]: The Physical Functioning (PF) scale describes whether health limits the ability to perform physical activities. The Bodily Pain (BP) scale describes the extent to which normal work activities are hampered by pain. The General Health (GH) scale describes the person’s self-rated health. The Vitality scale (VS) captures ratings of energy level. The Social Functioning (SF) scale measures the impact of either physical or emotional problems on social activities. The Role Physical (RP) and Role Emotional scale (RE) denote physical and mental health-related role limitations, respectively. The Mental Health (MH) scale measures psychological distress and well-being. The eight subscales are calibrated to have an average of 50 and a standard deviation of 10 in the general the United States (US) population (norm-based scoring), making it possible to meaningfully compare scores across domains. The tool has been used in Indian population and has been validated in local language (Odia) [22, 29].

### Data analysis

Three more chronic diseases arose from the additional list and were added to the previous list of 18 thus totalling to 21 chronic diseases. We followed the prescribed guidelines for analysis of PHQ-9 towards diagnosing depression and a score of 10 or more was taken as a cut-off value for depression [30].

Analysis was carried out using sampling weight which was calculated taking account of the complex nature of the sample, i.e. different sampling fraction in each CHC or private facility and taking account of clustering by facility by using the ‘svy’ command in STATA (Version 12.0, Stata crop, TX). Because of two-stage design nature, the probability weight is calculated as f1f2, which means that the inverse of the sampling fraction for the first stage (i.e. using the formula N/n, where N = the number of elements in the population and n = the number of elements in the sample) is multiplied by the inverse of the sampling fraction for the second stage. Percentages in this paper are weighted for sample and cluster adjusted for multilevel sampling. Confidence intervals of prevalence were calculated and the independent t- test or ANOVA test was performed for univariate analysis. We defined ‘multimorbidity’ as the presence of any two or more co-occurring chronic or long-term diseases or conditions. Patterns (dyads and triads) were determined by employing a simple matrix approach, exhaustive analysis of all possible combination of two or three co-morbid conditions using a simple explorative method based on the frequency [31].

We included conditions which had a prevalence of more than 1% in the analysis, since the number of cases below this was negligible. Age was categorized into two groups, less than or equal to 50 years and more than 50 years. Both physical component score (PCS) and mental component score (MCS) was calculated across the age group. Data was tested for normality, homoscedasticity, and absence of multi-collinearity using P-P plot and variance inflation factors (VIFs) for linear regression analysis. We estimated the factors associated with both PCS and MCS using linear regression analysis overall and the model-fit was evaluated by the F-value (< 0.05). To test the difference, we described each component score for major patterns of diseases in both the age group. A correlation matrix was developed for HRQoL, count of the chronic conditions and severity burden score. As we had multiple hypothesis to test, the expected *p* value was suspected to have high false discovery rate. In order to adjust the multiple testing problem, the adjusted p value was calculated using ‘Benjamini-Hochberg’ method [2, 32].

## Results

Out of the 1675 primary care patients approached, 1649 agreed to participate and were interviewed; 921 were men and 728 were women representing 6128 men and 4682 women after considering sampling weight. The average age of the sample was 44.0 years [95% CI: 43.2–44.9] and men’s [Mean 44.60, 95%CI: 43.50–45.70] age was slightly higher than women’s [Mean 43.30 95%CI: 42.00–44.50]. Even though nearly equal numbers of patients were recruited from both public and private facilities, weighted analysis shows slightly more than 2/3rd of the patients were recruited from public health facilities during their visits. Table 1 describes the socio-demographic distribution of the study participants.

**Table 1:** Socio-demographic distribution of the patients attending facilities

Characteristics.		Total (*n* = 1649) (Weighted %)	Percentage with multimorbidity (>2conditions) Weighted % [95%CI]	Mean PCS [95% CI]	Mean MCS [95% CI]
Age Group (in years)	18–29	373[22.6]	5.8[1.99–9.6]	44.21 [43.57–44.85]	43.65 [42.64–44.66]
	30–39	297[18.1]	22.2[15.1–29.4]	43.57 [42.84–44.29]	44.59 [43.47–45.71]
	40–49	346[20.5]	24.3[17.7–30.9]	43.12 [42.55–43.69]	43.41 [42.46–44.35]
	50–59	266[16.7]	36.2[27.9–44.5]	43.56 [42.86–44.26]	43.09 [41.98–44.20]
	60–69	236[14.6]	36.9[28.1–45.8]	43.49 [42.61–44.37]	43.92 [42.56–45.27]
	≥70	131[07.5]	44.4[33.0–55.8]	42.92 [41.73–44.12]	43.30 [41.50–45.11]
Gender	Men	921[55.8]	25.1[22.1–28.0]	43.61 [43.23–44.00]	43.48 [42.88–44.08]
	Women	728[44.2]	32.5[29.0–35.9]	43.50 [43.02–43.97]	43.96 [43.21–44.71]
Place of living	Rural	1493[90.4]	25.5[23.2–27.8]	43.56 [43.25–43.88]	43.55 [43.05–44.04]
	Urban	156[9.6]	28.5[27.8–29.3]	43.55 [41.61–44.49]	45.01 [43.38–46.64]
Ethnicity	Aboriginal	471[28.0]	27.7[26.3–29.2]	44.30 [43.71–44.88]	43.66 [42.76–44.55]
	Non-aboriginal	1178[71.4]	28.5[27.6–29.4]	43.27 [42.93–43.62]	43.70 [43.15–44.26]
Socio-economic status	Below poverty line	1035[61.6]	28.8[27.8–29.7]	43.59 [43.21–43.97]	44.04 [43.45–44.63]
	Above poverty line	601[38.4]	27.5[26.2–28.8]	43.52 [43.03–44.00]	43.14 [42.35–43.92]
Schooling	No School	642[38.1]	35.0[33.7–36.3]	45.33 [44.14–46.52]	44.03 [42.35–45.70]
	Primary completed	514[30.7]	28.3[27.1–29.5]	43.93 [43.42–44.43]	41.54 [40.85–42.23]
	Secondary and above	493[31.1]	20.1[19.6–21.1]	43.76 [43.16–44.36]	43.55 [42.61–44.49]
Marital Status	Currently married	1321[79.8]	29.3[28.5–30.1]	43.49 [43.17–43.81]	43.39 [42.88–43.89]
	Currently not married	328[20.2]	24.3[22.0–26.6]	43.83 [43.03–44.63]	44.88 [43.69–46.07]
Facility	Public	849[61.0]	28.1[27.1–29.1]	43.54 [43.13–43.96]	43.23 [42.60–43.96]
	Private	800[39.0]	28.6[27.5–29.7]	43.59 [43.17–44.00]	44.41 [43.72–45.11]
	Non multimorbidity	1082[71.7]		43.69 [43.35–44.03]	44.52 [43.96–45.08]
	Multimorbidity	567[28.3]		43.23 [42.62–43.84]	41.58 [40.74–42.43]
Total		1649[100]	28.3[25.9–30.7]	43.56 [43.26–43.86]	43.69 [43.22–44.16]

*PCS* Physical component score, *MCS* Mental component score

Around 28.30% (95% CI: 25.90–30.70) had multimorbidity while more than one-half (54.7%) had at least one chronic condition. Leading chronic conditions in men were acid peptic disease (28.40%), hypertension (15%), arthritis (13.40%), chronic back pain (10.60%) and diabetes (6.70%) whereas for women the most prevalent conditions were acid peptic disease (33.70%), hypertension (18.20%), arthritis (17.90%), chronic back pain (13.10%) and visual impairment (6.80%) were found to be leading. Details of the distribution of pattern of chronic conditions can be found in a previous paper [20].

Mean physical component score (PCS) and mental component score (MCS) of QoL in the study population was 43.56 [95% CI: 43.26–43.86] and 43.69 [95% CI: 43.22–44.16], respectively. Patients with multimorbidity reported poorer mean PCS [43.23, 95% CI: 42.62–43.84] and MCS [41.58, 95% CI: 40.74–42.43] compared to patients without multimorbidity (Table 1).

The effect of multimorbidity on SF-12 PCS and MCS is displayed in Table 2. In the final model this effect was adjusted for age, sex, place of residence, educational level, social class, and facility visit, separately for patients aged less than 50 years, more than and equal to 50 years and overall. Among patients aged less than 50 years, presence of multimorbidity decreases the MCS by 2.63 units [95% CI: − 3.89 to − 1.32], compared to non-multimorbids. In older adults (50 years or more), both PCS and MCS was negatively associated with multimorbidity, which reduces by 0.99 and 2.79 units, respectively after adjusting for other variables. A similar association was seen in an adjusted model for the whole study population, where multimorbidity had more inverse impact on MCS [regression coefficient: -2.76, 95% CI − 3.77 to − 1.75] compared to PCS [regression coefficient: -0.28, 95% CI − 0.98 to − 0.04] (Table 2).

**Table 2:** Linear regression for QoL (PCS and MCS) across the age group

		Less than 50 year (*n* = 1103)		More than 50 year (*n* = 546)		Overall (n = 1649)	
		PCS Adjusted coefficient [95%CI] Constant =43.32	MCS Adjusted coefficient [95%CI] Constant = 44.92	PCS Adjusted coefficient [95%CI] Constant = 45.21	MCS Adjusted coefficient [95%CI] Constant =42.12	PCS Adjusted coefficient [95%CI] Constant =43.15	MCS Adjusted coefficient [95%CI] Constant =44.23
Multimorbidity	No	Reference	Reference	Reference	Reference	Reference	Reference
	Yes	0.15 [− 0.66 to 0.98]	− 2.63 [− 3.89 to − 1.37]*	− 0.99 [− 2.19 to − 0.02]*	− 2.79 [−4.46 to − 1.11]*	− 0.28 [− 0.98 to − 0.04]*	− 2.76 [− 3.77 to − 1.75]*
		0.708	0.000	0.023	0.001	0.03	0.0001

*significant false discovery rate adjusted *p* value < 0.05, adjusted for Age, Gender, Caste, Education, Income, Marital status, place of living and Facility

For analysis survey command ‘svy’ was used

**Fig. 1:**
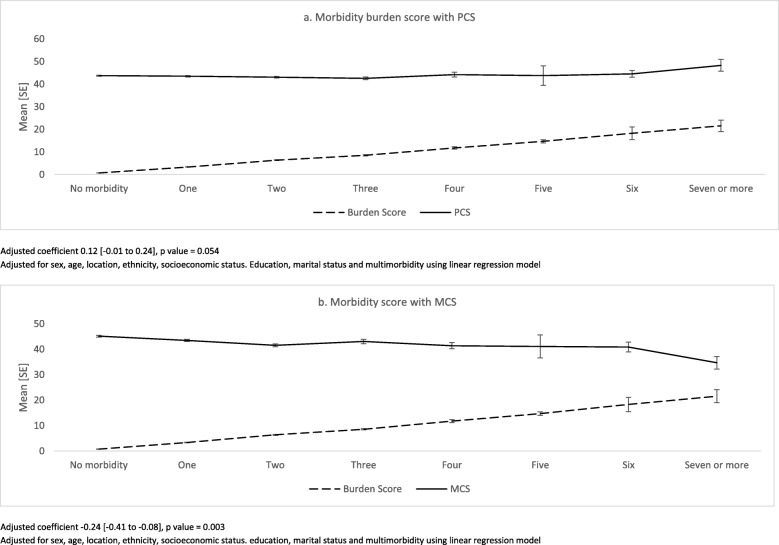
Relationship between burden score and QoL across number of chronic conditions (adjusted linear regression model). Adjusted coefficient 0.12 [− 0.01 to 0.24], p value = 0.054. Adjusted for sex, age, location, ethnicity, socioeconomic status. Education, marital status and multimorbidity using linear regression model. Adjusted coefficient − 0.24 [− 0.41 to − 0.08], p value = 0.003. Adjusted for sex, age, location, ethnicity, socioeconomic status. Education, marital status and multimorbidity using linear regression model

Adjusted linear regression models for eight HRQoL-components with disease count are described in Additional file 1: Table S1. RP (Role Physical), BP (Bodily Pain), SF (Social Functioning), RE (Role Emotional) and MH (Mental Health) subscales were found to be negatively associated with the count of chronic conditions. Figure 1 depicts the relationship of severity burden score with PCS and MCS, across the morbidity count. After adjusting for other variables, severity score was found to be negatively associated with MCS [regression coefficient: -0.24, 95% CI − 0.41 to − 0.08], whereas no significant association was seen with PCS. (Additional file 1: Table S1).

Within multimorbidity, physical QoL was seen to be better among those with no formal education and lower education, and mental QoL was inversely associated with illiteracy and lower education after adjusting for other socio-demographic variables (Table 3).

**Table 3:** Multivariate regression model for factors associated with QoL (PCS and MCS) in multimorbidity population

Variables	Categories	PCS Adjusted coefficient [95%CI] Constant =43.15	MCSAdjusted coefficient [95%CI] Constant =41.36
Gender	Men	Reference	Reference
	Women	0.43 [− 0.97 to 1.84]	0.74 [−1.13 to 2.66]
Place of living	Urban	Reference	Reference
	Rural	−0.80 [− 2.69 to 1.09]	− 0.74 [− 3.82 to 2.34]
Ethnicity	Aboriginal	Reference	Reference
	Non-aboriginal	1.43 [−0.19 to 3.06]	−0.89 [− 3.12 to 1.33]
Education	Secondary and above	Reference	Reference
	Illiterate	1.84 [0.14 to 3.54]*	−2.71 [−5.21 to −0.24]*
	Primary completed	1.23 [−0.21 to 2.67]	− 3.43 [−5.54 to −1.32]*
Socio-economic status	Below poverty line	Reference	Reference
	Above poverty line	−0.24 [−1.56 to 1.08]	0.23 [−1.60 to 2.06]
Marital status	Married	Reference	Reference
	Unmarried	0.42 [−1.96 to 2.81]	−0.44 [− 3.63 to 2.76]
Facility	Public	Reference	Reference
	Private	0.69 [−0.42 to 1.81]	−0.38 [−1.95 to 1.20]
Age group (years)	18–29	Reference	Reference
	30–39	−0.26 [−2.71 to 2.20]	4.62 [− 0.38 to 9.62]
	40–49	−1.14 [−3.36 to 1.07]	2.84 [− 2.10 to 7.79]
	50–59	−0.56 [− 2.92 to 1.80]	1.67 [− 3.32 to 6.66]
	60–69	− 0.95 [− 3.37 to 1.47]	3.05 [− 2.06 to 8.17]
	≥70	− 0.86 [− 3.53 to 1.81]	2.68 [−2.67 to 8.05]

*false discovery rate adjusted *P* value < 0.05

Table 4 describes the multimorbidity typology-related prediction of QoL adjusted for other variables. Among single chronic conditions, arthritis, chronic lung diseases, chronic back pain, epilepsy and depression are negatively associated with PCS, whereas, arthritis, hypertension, acid peptic disease, vision problem, epilepsy, tuberculosis, filariasis and depression had inverse relationships with MCS. Presence of arthritis with chronic back pain and vision impairment had poorer both PCS and MCS after adjusting for socio-demographic variables. Among triads, coexistence of acid peptic disease, hypertension and chronic back pain had adjusted coefficient of − 1.45 [95% CI − 3.23 to − 0.33], arthritis + hypertension + chronic back pain and arthritis + chronic back pain + chronic lung disease had negative adjusted coefficient for PCS. Hypertension and diabetes with arthritis and acid peptic diseases were found to be negatively related with MCS (Table 4).

**Table 4:** linear regression model for PCS and MCS for typology of multimorbidity

	PCS	MCS
	Coefficient [95% CI]	Coefficient [95% CI]
Arthritis	− 0.93 [− 1.81 to − 0.05]*	− 0.69 [− 2.02 to − 0.03]*
Diabetes	0.39 [− 1.10 to 1.90]	1.12 [− 0.69 to 2.93]
Hypertension	0.09 [− 0.68 to 0.86]	−2.09 [− 3.23 to − 0.95]*
CLD	−1.56 [− 3.03 to − 0.10*	−0.48 [− 3.40 to 2.45]
APD	0.15 [− 0.50 to 0.80]	−2.11 [− 3.11 to − 1.10]*
CBA	−0.50 [− 1.51 to − 0.05]*	−0.21 [− 1.71 to 1.29]
Vision problem	− 0.01 [− 1.46 to 1.43]	−2.92 [−4.55 to − 1.30]*
Deafness	0.89 [− 2.87 to 4.66]	− 1.94 [−6.43 to 2.54]
Epilepsy	− 2.85 [− 4.56 to − 1.14]*	−3.26 [− 6.50 to − 0.03]*
Alcohol problem	0.75 [− 2.68 to 4.18]	−2.82 [− 5.37 to − 0.28]*
Thyroid	0.89 [−1.76 to 3.54]	− 0.06 [− 5.03 to 4.89]
Tuberculosis	− 1.69 [− 3.91 to 0.52]	−3.84 [− 6.25 to − 1.44]*
Filariasis	1.31 [− 1.40 to 4.04]	− 3.44 [− 5.03 to − 1.84]*
Depression	3.59 [0.68 to 6.50]*	−6.77 [− 12.51 to − 1.02]*
Hypotension	−1.79 [− 4.01 to 0.41]	−0.58 [− 4.51 to 3.34]
APD + HTN	−1.39 [− 2.65 to − 0.13]*	1.55 [− 0.29 to 3.40]
APD + ART	− 0.0 [− 1.26 to 1.06]	0.50 [−1.26 to 2.26]
APD + CBA	0.26 [−0.83 to 1.35]	− 0.03 [− 1.85 to 1.78]
APD + VI	0.09 [− 1.66 to 1.86]	−0.04[− 2.13 to 2.04]
APD + DI	−0.31 [− 2.21 to 1.59]	1.07 [− 2.14 to 4.30]
APD + CLD	0.52[− 2.73 to 3.76]	2.22 [−1.43 to 5.89]
APD + DF	− 1.81 [− 4.72 to 1.08]	3.19 [− 2.60 to 9.00]
HTN + ART	− 0.87 [− 2.74 to 1.01]	1.11 [−1.43 to 3.67]
HTN + DI	0.63 [− 125 to 2.52]	−1.28 [− 2.74 to − 0.30]*
HTN + CBA	−1.11[− 2.72 to 0.49]	−0.06 [− 2.52 to 2.38]
HTN + VI	−0.47 [− 2.98 to 2.04]	−1.84 [− 4.14 to 0.45]
ART + CBA	− 0.65 [− 1.09 to − 0.09]*	−0.79 [− 1.27 to − 0.06]*
ART + VI	−0.31 [− 1.13 to − 0.06]*	−0.33 [− 1.72 to − 0.05]*
ART + DI	0.07 [− 2.91 to 3.05]	0.80 [− 3.73 to 5.34]
APD + HTN + CBA	−1.45 [−3.23 to − 0.33]*	−0.18 [− 2.90 to 2.35]
APD + HTN + DI	−1.28 [− 3.85 to 1.27]	−1.80 [− 4.68 to − 0.09]*
HTN + ART+CBA	−0.89 [− 3.19 to − 0.32]*	1.23 [− 2.16 to 4.63]
HTN + ART+DI	2.75 [− 0.10 to 5.61]	−3.14 [− 6.32 to − 0.03]*
APD_ART_VI	−0.56 [− 2.89 to 1.75]	−0.60 [− 3.36 to 2.15]
ART+CBA + CLD	−3.13 [− 9.24 to − 0.09]*	2.66 [− 3.37 to 8.71]
ART+CBA + VI	−0.54 [− 3.26 to 2.17]	0.23 [− 3.74 to 4.20]
APD + CBA + VI	−1.46 [− 3.81 to 0.88]	0.95 [− 2.87 to 4.77]

*Significant false discovery rate adjusted p value < 0.05; adjusted for age, sex, caste, education, income, marital status, religion, multimorbidity, place of living and Facility

*APD* Acid peptic disease, *HTN* Hypertension, *ART* Arthritis, *DI* Diabetes, *CBA* Chronic backache, *VI* Visual impairment, *DF* Deafness, *CLD* Chronic lungs disease

## Discussion

Multimorbidity, the concurrent presence of two or more chronic medical conditions, is consistently associated with a series of adverse health outcomes, and most notably evokes a negative effect on quality of life and wellbeing [8]. An important objective of any health care system is to increase the span of life years while maintaining an optimal quality of life, thus making HRQoL a primary concern of patients, clinicians, and policy interest [13].It is largely unknown how the HRQoL is influenced by multimorbidity among primary care population in India, with no studies reporting the effect of disease severity and pattern [16]. We undertook the first-ever study to assess HRQoL of Indian primary care patients with multimorbidity and the factors contributing to its impairment with the aim of providing insights towards designing multimorbidity-specific interventions. This is particularly relevant since in India, as in other countries, the clinical guidelines are usually configured to manage single diseases and rarely account for multimorbidity [33] and the therapeutic plan is mostly additive rather than integrative.

We found an inverse association between multimorbidity and HRQoL in consistence with that reported from both high and low income settings [34, 35]. Recent studies from countries like Brazil and China have reiterated the linear negative relationship between number of chronic conditions and HRQoL [36, 37]. The increase in number of chronic conditions in an individual lead to higher healthcare utilisation and expenditure, and impairs both physical and mental health, thus resulting in poorer QoL [38–40].

In our study, multimorbidity was found to adversely affect both physical and mental QoL, among patients aged 50 years or more after controlling for confounding variables. The deteriorating effect of multimorbidity in elderly age groups has been well established across multiple geographic and practice settings [41, 42]. This finding has two major corollaries in the Indian context. First, it directs the attention towards age-specific care planning for multimorbidity. Second, it indicates the potential role of National Program for the Care of the Elderly (NPCE) in the context of multimorbidity. The program has been broadly designed to improve the overall health and wellbeing of elderly population in India. With the given impact of multimorbidity on wellbeing, the geriatric program has to construe its guidelines responsive to the diverse care needs [43].

To date, a major share of multimorbidity research has been carried out in ageing populations, thus resulting in deficient understanding of QoL in midlife population [44] In our study, the mental component of QoL was found to decline in people aged less than 50 years having multimorbidity. The impairment of mental QoL in a relatively younger and productive age group is concerning and indicates and increased level of psychological problems among these population groups. Considering the findings on magnitude of multimorbidity in younger population, future studies should specifically investigate the QoL and its correlates in this particular demographic group.

Our finding provides ample reasoning for a collaborative patient-centred model to address the dual physical and mental care needs in multimorbidity. However, in India, the National Mental Health Program operates vertically separate from the NCD control program [45]. Thus, there could be a scope for collaborative entwining of the two programs at the primary care level. An implementation trial could be undertaken to test such integrated model within the newly created “Health and Wellness Centres” in primary care settings [46, 47].

We could not find a significant difference between gender and HRQoL. Many previous studies have also demonstrated sparse evidence on sex specific differences in quality of life in multimorbidity [48].

Socio-economic factors are known to exert significant influence on multimorbidity prevalence and outcome [49–51]. In our study, lower education was inversely associated with mental QoL and positively associated with physical QoL score after adjusting for other variables. A systematic review has documented the universal effect of literacy on multimorbidity [52]. Access to education has been identified as one of the key social determinants of health (SDH) by the World Health Organisation [53]. Future multimorbidity work may explore how social determinants like literacy mediate the effect of multimorbidity on quality of life.

It is well established that QoL often decreases with increase in the number of chronic conditions. Our findings too resonate with this global observation. However, multimorbidity studies in high income countries have identified severity of the condition as an important driver of QoL than simple count alone [54]. We found increase in severity score to be negatively associated with mental QoL particularly. The severity was measured by the level of activity limitation, which is a proxy indicator of functional impairment affecting PCS [27] As most of the chronic diseases causing direct physical impairment such as osteoarthritis and lung diseases are age related, a non-significant association of multimorbidity with PCS is justified after adjustment of age and other factors. However, its inverse association with MCS after controlling for other variables is an interesting finding, which merits further investigation. Given the larger impact of severity on the mental QoL, it is necessary to identify those individual chronic conditions contributing to higher severity score.

Our study made a further contribution by investigating the impacts of different multimorbidity patterns on QoL. Most frequently occurring multimorbidity patterns were identified and their association with QoL was estimated while controlling for other socio-demographic variables. Most of the available studies on multimorbidity patterns and their association with QoL are confined to the elderly population (≥65 years); thus, it is difficult to draw comparison of our results [16]. In our study, patterns having arthritis have impaired PCS of QoL after controlling for other variables. Similar findings have been reported from other studies, where presence of osteoarticular disease had an inverse association with PCS [55]. Among triads, presence of arthritis and chronic back pain had a similar inverse significant association. This could be explained by the limiting effect of the disease on physical functioning, body function, self-care ability, social adaptability. Besides, patients with hypertension, diabetes, and lung diseases were found to have a decline in mental QoL. Conditions like hypertension and diabetes are related with stress, and coexistence of these conditions might have accentuated the impact on psychological wellbeing owing to chronic care demand [56]. Thus, identification of specific patterns could help to understand the impact of multimorbidity on different dimensions of QoL and prioritise patient groups for improving QoL.

### Strengths and limitations

The present study has certain limitations worth mentioning. The first refers to the cross-sectional design, where respondents were analysed at a given time and that cannot establish a causal relationship. Second, the information was collected through self-report and omissions may have occurred. To address this, the presence of chronic diseases was confirmed by the use of medicines and information on prescriptions. Third, we did not record the duration of the chronic illness and health care expenditure which might have had some influence on HRQoL. Nevertheless, our study using a validated questionnaire with a comprehensive list of chronic conditions captured the first ever information on the effect of number of chronic diseases, and pattern and severity of multimorbidity on health related quality of life. The use of a representative sample of the primary care population, an iterative process for study tool development and validation, training of interviewers, and the fact of being the first Indian study to investigate the association between chronic disease multimorbidity and HRQoL in adult primary care patients are the key strengths.

### Implications for policy, practice and research

Our study provides the first ever report on health related quality of life in multimorbidity in an Indian primary care population. We found an inverse relationship between the number of chronic conditions and HRQoL, for both physical and mental domains. However, the number of chronic diseases and severity correlate better with quality of well-being than count alone. Thus, assessment of multimorbidity burden should include severity along with the number of individual chronic conditions.

Given the significantly high impairment of physical and mental QoL in patients with multimorbidity aged above 50 years, it is important to ascertain the care needs and design plans specific for this age group. This necessitates a functional amalgamation between three national level programs in the country i.e. geriatric care, NCD Control and mental health towards an horizontally integrated care model for elderly patients with multimorbidity under the umbrella of primary care.

## Conclusion

Specific combinations of chronic conditions appear to have differential effects on HRQoL. The amplified effect of musculoskeletal condition on QoL has strong policy implications since the extant national NCD control program is yet to include arthritic conditions under its ambit. While designing clinical management guidelines for multimorbidity, priority is to be accorded to clusters having musculoskeletal diseases tailored therapeutic plans. These need to be tested in primary care practice. It may be prudent to include severity as an intermediary patient reported outcome measure to evaluate the effectiveness of these interventions. Future research should consider pursuit of a primary care cohort of multimorbidity, follow the trajectory and analyse the interactions of conditions. This would provide a strong methodological setting to investigate the long-term effects of strategies to improve HRQoL in different subgroups and inform primary care policy.

## Additional file

Additional file 1: **Table S1.** Linear regression model for eight components of SF-12 for number of chronic conditions. (DOCX 14 kb)

## Data Availability

The datasets used and/or analysed during the current study are available from the corresponding author upon valid request.

## Abbreviations

ANOVA: Analysis of covariance
BP: Bodily pain
CHC: Community Health Centre
CI: Confidence interval
GH: General Health
HRQoL: Health related quality of life
LMIC: Low and Middle Income Countries
MAQ –PC: Multimorbidity assessment questionnaire-primary care
MCS: Mental component score
MH: Mental Health
NCD: Non communicable diseases
PCS: Physical component score
PF: Physical functioning
PHQ − 9: Patient Health Questionnaire-9
QoL: Quality of life
RP: Role physical
SDH: Social Determinants of Health
SE: Role social emotional
SF: Social functioning
US: United States
VIF: Variation inflation factor
VS: Vitality scale
