# Supplementary material for: Health related quality of life in multimorbidity: a primary-care based study from Odisha, India
Source: Health Qual Life Outcomes. 2019 Jul 5;17:116. doi: 10.1186/s12955-019-1180-3 (PMC6612103; doi:10.1186/s12955-019-1180-3)
Supplement: Supplementary file 1 — Table S1. Linear regression model for eight components of SF-12 for number of chronic conditions. (DOCX 14 kb) [file 12955_2019_1180_MOESM1_ESM.docx]

Additional File-1

Table -1 linear regression model for eight components of SF-12 for number of chronic conditions

| Chronic conditions | Physical functioning | Role Physical | Body Pain | General health | Vitality | Social  Functioning | Role  Emotional | Mental Health | PCS | MCS |
| --- | --- | --- | --- | --- | --- | --- | --- | --- | --- | --- |
| None | Reference | Reference | Reference | Reference | Reference | Reference | Reference | Reference | Reference | Reference |
| One | -0.03  [-0.14 to 0.08] | -0.11  [-0.21 to -0.01]* | -0.08  [-0.22 to 0.05] | -0.03  [-0.13 to 0.07] | 0.01  [-0.10 to 0.14] | -0.14  [-0.27 to -0.01]* | -0.18  [-0.31 to -0.03]* | -0.01  [-0.13 to 0.10] | -0.54  [-1.32 to 0.25] | -0.85  [-2.07 t0 0.38] |
| Two | -0.15  [-0.31 to 0.01] | -0.22  [-0.37 to -0.07]* | -0.26  [-0.45 to -0.07]* | 0.01  [-0.15 to 0.16] | 0.08  [-0.10 to 0.26] | -0.46  [-0.67 to -0.25]* | -0.37  [-0.57 to -0.17]* | -0.8  [-0.23 to -0.07]* | -1.27  [-2.44 to -0.11]* | -2.20  [-3.82 to -0.58]* |
| Three or more | -0.13  [-0.36 to 0.09] | -0.31  [-0.53 to -0.10]* | -0.04  [-0.29 to 0.20] | -0.07  [-0.32 to 0.17] | 0.17  [-0.12 to 0.47] | -0.59  [-0.92 to -0.26]* | -0.38  [-0.66 to -0.10]* | 0.17  [-0.05 to 0.38] | -1.55  [-3.24 to 0.13] | -1.26  [-3.64 to 1.11] |

*significant p value adjusted for sex, age, location, ethnicity, socioeconomic status, education, marital status, burden score and multimorbidity
